# Comparative Effectiveness of Teaching Obstetrics and Gynaecological Procedural Skills on Patients versus Models: A randomized trial

**DOI:** 10.12669/pjms.344.15521

**Published:** 2018

**Authors:** Shereen Zulfiqar Bhutta, Haleema Yasmin

**Affiliations:** 1 Prof. Shereen Zulfiqar Bhutta, Department of Obstetrics and Gynaecology, Jinnah Postgraduate Medical Centre, Karachi, Pakistan; 2 Dr. Haleema Yasmin, Department of Obstetrics and Gynaecology, Jinnah Postgraduate Medical Centre, Karachi, Pakistan

**Keywords:** Skills, Simulation, Models, Mannequins, Assessment

## Abstract

**Objective:**

To compare the effectiveness of learning procedural skills on patients versus mannequins and models.

**Methods:**

Seventy four interns from two consecutive batches at the Department of Obstetrics and Gynaecolgy Unit-I at Jinnah Postgraduate Medical Center Karachi participated in the study between April and September 2014. Five basic skills; taking a cervical (Pap) smear, intrauterine contraceptive device insertion, manual vacuum aspiration, making/ suturing an episiotomy and active management of the third stage of labour were identified. Interns were randomly allocated to two training groups (Group-1 and 2 of thirty eight and thirty six trainees respectively), with Group-I received training on the five procedural skills on models and mannequins for four weeks while Group-II trained on patients initially. After an evaluation at four weeks the groups crossed over with a final evaluation at eight weeks. The evaluation was through identical objective structured assessment of technical skills on models and mannequins for both groups with standard checklists.

**Results:**

There was no significant difference in skills between the two groups at the four weeks assessment. However at the end of training, Group-1 trainees performed significantly better than Group 2 with higher overall tests scores (86.7 ± 2.7 versus 80.4 ± 4.8, p< 0.001). This difference was more marked in skills of intrauterine contraceptive device insertion, making and suturing an episiotomy and active management of third stage of labour.

**Conclusion:**

Our findings suggest that simulations using models and mannequins for developing procedural skills can be readily incorporated in training programs with potential benefits for teaching infrequently performed or more difficult procedures. Our data suggest potential benefits of initiation of trainings on simulations and mannequins followed by human subject exposure.

## INTRODUCTION

Interns in Obstetrics and Gynaecology (Ob Gyn) have traditionally been taught common procedural skills on human subjects, usually patients.^1^ In Pakistan, at least in the public sector, it has been possible to do this so far because there is no dearth of patients ‘willing’ to facilitate this, a reality of receiving care in public sector hospitals. With increasing awareness among patients about their autonomy and the need for informed consent for multiple examinations, alternative teaching strategies are needed.^2^ The need for standardization and practice is an important consideration to seek alternatives to the approach of “see one, do one, teach one”.^3^ necessitating alternative strategies. While teaching procedural skills to trainees using simulations and mannequins was first initiated as far back as the 17th century,^4^ The concept has mainly evolved over the last few decades.^5^ While simulations and teaching of procedural skills on models have been successfully used in various settings^6^-^9^, their widespread incorporation within curricula in low and middle income settings is sporadic and largely limited to institutions in high income settings.^10^,^11^

In the practice of Obstetrics and Gynaecology in conservative societies such as in Pakistan, the use of model or mannequin-based simulations is especially promising because repeated examinations or procedures on female patients is difficult.^12^ The present study was planned with the objective of determining whether utilization of mannequins and models for training interns in Ob Gyn in a structured program would result in knowledge and competencies comparable to those training on human subjects primarily, and up to the required standards for certification.

## METHODS

We undertook a mixed methods evaluation for two alternative teaching strategies among fresh medical graduates undertaking basic training in Ob Gyn at JPMC. Altogether eighty six trainees from two consecutive batches of fresh medical graduates from Sindh Medical College entering the basic internship program at the Department of Ob Gyn JPMC Unit-1 between the months of April and September 2014 were eligible for inclusion in the study. Of these, twelve did not participate as they were unable to undertake uninterrupted training. All trainees had received five years of undsrraduatemedical education and successfully passed the final certification examination as well as the entrance examination of the JPMC for clinical training. Given concerns about training house officers solely on a new simulation-based process, a pragmatic design was chosen which included the traditional training approach for all trainees. A pragmatic sample size was selected for this evaluation as no prior data existed to undertake a formal sample size and power estimation. Five basic Ob Gyn skills that interns are expected to perform competently were identified *a priori*. These consisted of taking a cervical (Pap) smear, Intrauterine Contraceptive Device (IUCD) insertion, manual vacuum aspiration, making and suturing an episiotomy and Active Management of the Third Stage of labour (AMTSL).

**Fig.1:**
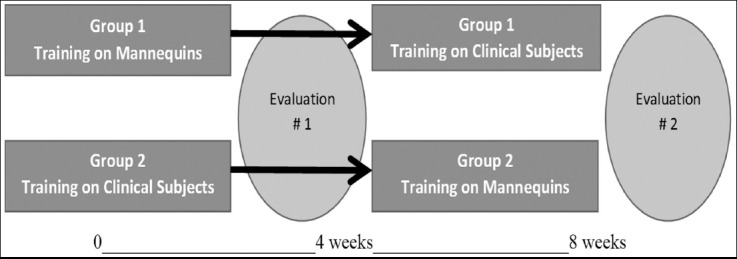
Training period.

Seventy four interns provided informed consent and were randomly allocated to two groups. Group-1 consisted of thirty eight participants who were given an initial opportunity to learn the five procedural skills on models and mannequins only with postings in the inpatient wards. Group-2 had 36 participants who were initially posted in the Labour ward, Gynaecological emergency services, and Operation Theater, Outpatient and Family Planning clinics. This group learnt and developed the five selected procedural skills on actual patients, as per prevalent practice. After four weeks the groups crossed over for training with clinical subjects or mannequins, and thus the main difference between the two groups was in the sequencing of training (Fig.1). The opportunities for interaction with patients and participation in other educational activities of the department were the same for both groups of trainees.

A pre-assigned set of clinical supervisors blinded to the group allocation, evaluated the procedural skills of the trainees on models and mannequins using standardized tools, the Objective Structured Assessment of Technical Skills (OSATS) at four weeks and eight weeks. Checklists with global rating scales were used to obtain quantitative scores. Following the crossover at four weeks, training on patients and mannequins was continued as previously. The same evaluation using OSATS on mannequins and models was repeated near end of training and scores were used to identify shortcomings in skills and remedial training was imparted by the supervisors.

## RESULTS

The performance of the trainees after four weeks of training and did not reveal any significant differences in skills between the two groups with the exception of higher performance of Group 1 trainees in the performance of episiotomy are summarized in Table-I.

**Table-I:** Group performance on procedures in intent-to-treat analysis (midway of training) Data as mean score ± SD

Procedures evaluated	Group 1	Group 2	P value

	N = 37	N = 36	
Manual Vacuum aspiration	14.9 ± 1.7	14.9 ± 1.9	0.996
Obtaining Cervical smear	13.3 ± 1.5	13.1 ± 2.6	0.970
IUCD insertion	11.6 ± 1.9	12.3 ± 2.6	0.219
Making/suturing episiotomy	15.8 ± 1.6	14.8 ± 2.7	<0.05
Active management of 3^rd^ stage of labour	14.4 ± 2.1	14.7 ± 2.5	0.627
Overall score	69.8 ± 4.4	70.0 ± 6.8	0.888

However at assessment at the end of week 8 (Table-II), Group-1 trainees performed significantly better than Group-2 trainees in most aspects of skills acquisition and with higher overall tests scores (86.7 ± 2.7 versus 80.4 ± 4.8, p< 0.001). The interns who had trained initially on models and mannequins and thereafter on actual patients, scored significantly higher overall in assessment. This difference was more marked in skills of IUCD insertion, making and suturing an episiotomy and AMSTL.

**Table-II:** Group performance on procedures in intent-to-treat analysis (end-line) Data as mean score ± SD

Procedures evaluated	Group 1	Group 2	P value

	N = 37	N = 36	
Manual Vacuum aspiration	16.7 ± 1.4	16.9 ± 1.3	0.505
Obtaining Cervical smear	15.6 ± 1.5	15.9 ± 1.7	0.438
IUCD insertion	16.2 ± 1.5	15.3 ± 1.7	0.018
Making/suturing episiotomy	18.3 ± 0.9	16.2 ± 2.3	<0.0001
Active management of 3^rd^ stage of labour	18.1 ± 1.1	16.2 ± 1.7	<0.0001
Overall score	86.7 ± 2.7	80.4 ± 4.8	<0.0001

## DISCUSSION

Learning skills on patients the traditional way using the apprenticeship model is fraught with problems like lack of practice opportunities, anxiety for fear of causing harm and transgression of patient autonomy.^13^ Simulation-based training using models and mannequins is one such alternative, but is still viewed with skepticism. There is evidence that this approach can be incorporated in training programs without compromising on standards.^14^-^16^ In some instances using models alone or alongside patients has been shown to improve performance.^17^

A deterrent to this approach is the expense of acquiring and maintaining models and mannequins, especially in low resource settings. It is worth considering that one time investment is likely to pay for itself in the long run. Initially nonspecific generic models with well-trained motivated facilitators can get the training off the ground, ultimately leading to effective use of skills laboratories.^18^,^19^ Furthermore, low cost appropriate local alternatives are usually available and are even better at times. In this study too, all the models, mannequins and materials were produced locally at affordable cost.

Although the assessment did not show a significant difference between the final scores of those practicing on models or patients, those who had practiced initially on models followed by patients, had better overall assessment scores closer to the end of training. They also had greater confidence in their skills.

Simulation is preferably used to develop maintain and improve skills of health care providers until proficiency is achieved, without harming patients. There is evidence that those who train initially on models have a shorter learning curve^20^,^21^ and are better prepared for clinical practice.^22^ They understand the clinical implications of these skills better and have a higher adherence to protocols, this in turn would prepare them to deal with unexpected complications in actual clinical practice. Those Interns who had trained initially on models and then performed procedures on patients reported a higher comfort level than their contemporaries who had only practiced on patients. Residents’ “comfort” when performing procedures has been proposed as an alternative marker of competence in several studies.^23^,^24^

Interaction with patients is an integral part of patient doctor relationship. It is a skill that is equally important to the technical skills. If the entire training is limited to training on models and mannequins, this aspect is likely to suffer,^25^,^26^ so the two strategies need to supplement rather than replace each other.

The difference in assessment scores was noticeable for relatively more invasive procedural skills like IUCD insertion, making and suturing an episiotomy and AMSTL. Here interns in Group-1 scored higher than those in Group-2, suggesting that this approach might be even more suitable for relatively infrequently performed or more difficult procedures.^27^ The reason could be that the procedures could be practiced repeatedly on models at convenience and in accordance with the laid down protocols, without fear of causing harm or discomfort. This in turn would lead to fewer adverse events at time of real application and also equip the trainees to deal better with potential complications. In addition, this approach can be used more effectively and objectively for setting training standards and objective assessment with greater validity.^28^,^29^

There is a need to promote innovative, improved and more objective training strategies for doctors in addition to training on patients. For decades, the airline industry has been training pilots on aircraft simulators before entrusting them with lives of passengers.^30^ It is time we did the same for our trainees and the women under their care.

### Author’s Contribution

**SB:** Conceived, prepared project, data collection and processing, preparation of manuscript.

**HY:** Project implementation, data collection.
